# Long-Term Outcomes of Sleeve Gastrectomy Versus Gastric Bypass

**DOI:** 10.7759/cureus.72961

**Published:** 2024-11-04

**Authors:** Omar Alaidaroos, Azzam A Al Jaber, Abdulrahman A Al Jaber, Abdullah H Alshehri, Majed B Alkehaimi, Omar A Alsannat

**Affiliations:** 1 Surgery, AlMaarefa University, Riyadh, SAU

**Keywords:** gastric bypass, laparoscopy, long term effect, obesity, sleeve gastrectomy

## Abstract

Background: Extremely obese patients can benefit greatly from bariatric surgery, a common and successful therapeutic procedure for treating obesity and accompanying medical issues. Although sleeve gastrectomy and gastric bypass have already demonstrated their effectiveness in this demographic, long-term results were not stated in the literature. The purpose of this research is to examine the long-term outcomes of sleeve gastrointestinal surgery and gastric bypass.

Method: This retrospective, single-center study compares 100 patients aged 25 years and older who visited the gastrointestinal tract surgical unit at Dar El-Fouad Hospital in Cairo, Egypt, between January 1 and August 31, 2019, according to the inclusion and exclusion criteria. The patients underwent either a Roux-en-Y gastric bypass (RYGB-50%) or sleeve gastrectomy (SG-50%) for severe obesity. Follow-up occurred at one year and up to four years following surgery to collect information from the study subjects. Two tools were used to assess BMI, weight loss, complications after surgery, and incidence outcome of comorbidities after the two surgeries. Qualitative data were presented as number and percentage and frequency distribution tables, and every analysis was done at a significance value < 0.05.

Result: The average age of patients within the SG group was 43.02 ± 9.19 years, whereas the average age of patients within the RYGB group was 41.02 ± 11.06 years. In addition, 74% of patients were women in both procedures. The BMI mean of the SG group was 43.90 ± 5.78, the BMI mean of the RYGB group was 42.73 ± 5.12, and the main comorbidity in both techniques was joint pain. The mean BMI at one year was 29.70 kg/m^2^ after SG compared with 28.64 kg/m^2^ after RYGB. After four years, BMI was regained within the obese range in both techniques - 30.67 kg/m^2^ and 30.32, respectively. Fewer postoperative complications occurred in SG than in RYGB. RYGB was superior to SG in managing dyslipidemia (DL), hypertension (HT), type 2 diabetes (T2DM), joint pain, and gastroesophageal reflux disease (GERD).

Conclusion: There are no significant differences between the SG and RYGB in long-term outcomes regarding BMI before surgery and at follow-up, after four years, while there were statistically significant differences between them after four years than one year after surgery, and both groups showed a significant decrease in weight. However, RYGB shows improvement to some extent in comorbidities within follow-up period, including BMI, T2DM, HT, DL, HT, DL, GERD, and joint pain than SG, but with a higher rate of minor complications, while greater resolution of OSAS occurred in SG. Finally, at four years, there were no discernible variations in BMI between SG and RYGB because the patients' mean BMI was within the obese range once more.

## Introduction

The term “obesity” refers to an excess or abnormal buildup of body fat that can harm a person’s health. Its cause is multifactorial and complex due to the interaction of genes, lifestyle, environment, and emotional factors [1]. The worrisome rise in obesity, a global health concern, demonstrates the necessity of better therapies. Metabolic operations, including sleeve gastrectomy (SG) and Roux-en-Y gastric bypass (RYGB), have proven to be successful treatments [2]. In 2018, nearly 600,000 primary surgical bariatric surgeries were carried out globally, with RYGB and SG the most widely used procedures [3].

Globally, SG and gastric bypass are the most widely used bariatric operations [4]. For patients who are extremely obese, bariatric surgery is the only intervention that promotes significant and long-term weight loss. However, due to the lack of long-term bariatric surgery studies with adequate patient follow-up, the results of these procedures remain uncertain [5].

In SG surgery, five or six upper abdominal ports are used to reshape the stomach into a narrow, tubular structure [6], with two-thirds of the stomach being resected [7]. Because SG reduces stomach capacity and modifies gastric motility and hormonal levels (namely ghrelin and peptide YY), it induces weight loss [8,9].

Another operation for weight loss that limits the amount of food patients can eat is gastric bypass, commonly known as RYGB surgery. It is often performed laparoscopically or openly through a large abdominal incision to create a “Y”-shaped opening in the small intestine. The upper stomach is surgically sutured into a small, egg-sized pouch and then directly attached to the Roux limb [10].

Short-term data suggest that weight loss following SG is almost identical to that following bypass surgery but with a lower risk of perioperative complications. Furthermore, SG, which involves a 70% vertical gastric resection without intestinal bypass, is less technically demanding than bypass surgery. Additionally, preliminary findings indicate that SG is effective in managing type 2 diabetes and morbid obesity [11].

Although SG’s popularity has surpassed data from thorough comparative effectiveness research studies, there is still disagreement over the long-term safety and robustness of SG compared to bypass surgery [12]. The most significant drawbacks of SG are its irreversibility and the increased risk of gastroesophageal reflux disease (GERD), which can result in Barrett’s esophagus [13].

Recent randomized trials comparing the two operations and thorough systematic reviews support the application of SG [11,14,15]. The Swiss Multicenter Bypass or Sleeve Study aimed to demonstrate a 10% greater difference in weight loss between the two methods, whereas the goal of the sleeve versus bypass (SLEEVEPASS) study was to demonstrate an equivalent amount of weight loss. Although no conclusive findings could be drawn after long-term follow-up, these trials suggested that RYGB might be advantageous. The Oseberg experiment, which evaluated type 2 diabetes remission between the two procedures, showed that RYGB produced greater improvement in type 2 diabetes at the three-year follow-up [14].

There are concerns that SG may not be as effective as other procedures for long-term weight loss and type 2 diabetes remission due to unpredictable and inconsistent long-term outcomes [11,14,15].

In terms of weight loss and comorbidity resolution, the long-term outcomes of SG may be similar to those of RYGB. The results presented in this article include follow-up data at one year and four years.

## Materials and methods

Method

Research Design

The current investigation employed a retrospective, comparative, single-center design. Additionally, this study was conducted in compliance with ethical guidelines, and participants provided written informed consent.

Subjects and Setting

This study included 100 patients over 25 years old who visited the gastrointestinal tract surgical unit at Dar El-Fouad Hospital in Cairo, Egypt, between January 1 and August 31, 2019. Patients were included according to specific inclusion and exclusion criteria and underwent either an RYGB (50%) or SG (50%) due to extreme obesity. Follow-up occurred at one year and up to four years post-surgery.

Inclusion criteria: (1) every patient who underwent either an RYGB or SG at the gastrointestinal tract surgical unit between January 1 and August 31, 2019, and who satisfied the worldwide criteria for metabolic surgery eligibility, was invited to participate; (2) participants over 25 years old, with a BMI greater than 40 kg/m², and willing to participate and provide written consent; and (3) patients with a BMI over 35 kg/m² along with obesity-related comorbidities.

Exclusion criteria: (1) patients under 25 years old with a BMI of less than 40 kg/m²; (2) individuals who underwent bariatric procedures other than SG and RYGB; (3) patients with a symptomatic hiatal hernia; (4) patients with a history of significant abdominal surgery or metabolic conditions; (5) patients unable to provide informed consent or having difficulty understanding the surveys; and (6) patients unable to adhere to prearranged clinical follow-up.

Surgical Techniques

Based on previously published descriptions of protocolized surgical techniques, the two procedures were carried out laparoscopically [16]. SG was performed using a linear stapler (I-Drive with Tri-Staple cartridges, Medtronic, USA) inserted along the lesser curvature with a 40 French (Fr) bougie serving as a calibration tool. In the RYGB procedure, which began near the 3-5 cm prepyloric region, a 36 Fr bougie calibrated a 6 cm long gastric pouch. This was done using a linear stapler (I-Drive with Tri-Staple cartridges; Medtronic, Minneapolis, MN) with a 100 cm biliopancreatic limb and a 150 cm alimentary limb. The gastrojejunal anastomosis was calibrated at 2 cm during the execution of both anastomoses using a linear stapler (I-Drive with Tri-Staple cartridges). Unless technically impractical, both mesenteric defects were conventionally closed.

Data Collection

All data were gathered at baseline, one year, and four years post-surgery. Various questionnaires were also used, along with assessments of weight, blood tests, morbidity, and mortality [17,18].

Outcome and Comorbidity Resolution

Two tools were employed to evaluate BMI, weight loss, postoperative complications, and the incidence and resolution of comorbidities following the two surgeries.

At baseline (preoperative values) and one year and four years post-surgery, BMI was assessed as one of the anthropometric factors. Comorbidities associated with obesity, such as hypertension (HT), type 2 diabetes mellitus (T2DM), dyslipidemia (DL), GERD, and obstructive sleep apnea syndrome (OSAS), were improved or resolved, in addition to postoperative complications.

The resolution of T2DM was defined as plasma glucose below 110 mg/dL and glycated hemoglobin below 6.5% in the absence of hypoglycemic medication. Resolution of DL was defined as high-density lipoprotein cholesterol over 40 mg/dL, total cholesterol below 200 mg/dL, and fasting plasma triglycerides below 200 mg/dL, all without pharmaceutical therapy. Blood pressure below 135/85 mmHg, without the use of antihypertensive medication, was considered resolved HT. Resolution of GERD was defined as the absence of symptoms and the avoidance of medical intervention, equipment, or medication. Mild GERD symptoms were classified as those not present daily and controllable without the use of proton pump inhibitors.

Statistical analysis

Data entry and analysis were conducted using Statistical Product and Service Solutions (SPSS, version 22.0; IBM SPSS Statistics for Windows, Armonk, NY) software. Qualitative data were presented as numbers and percentages, with means, standard deviations, and frequency distribution tables. All analyses were performed at a significance level of < 0.05. Comparison of qualitative variables was carried out using the chi-square test.

## Results

Characteristics of the study subjects

Table 1 reveals that the average age of individuals who underwent SG is 43.02 ± 9.19, and the average age of patients who underwent RYGB is 41.02 ± 11.06. In addition, 74% of SG patients were women compared with 88% of RYGB patients. In addition, 50% were homemakers in the SG group compared with 72% in the RYGB group.

**Table 1:** Distribution of the patients regarding their characteristics (n = 100) SD = standard deviation

Item	SG (n = 50)	RYGB (n = 50)
Age (years) mean ± SD	43.02 ± 9.19	41.02 ± 11.06
Gender		
Male	13 (26%)	6 (12%)
Female	37 (74%)	44 (88%)
Occupation		
Homemaker	25 (50%)	36 (72%)
Freelancer	5 (10%)	3 (6%)
Employee	20 (40%)	11 (22%)

Table 2 reveals that the BMI mean of the sleeve group is 43.90 ± 5.78, and 40%, 32%, 20%, 18%, and 4% of them suffer from joint pain, hypertension, DM, dyslipidemia, OSAS, and GERD, respectively. However, the BMI mean of the RYGB group is 42.73 ± 5.12, and 30%, 26%, 20%, 16%, 10%, and 8% of them suffer from joint pain, DM, hypertension, OSAS, GERD, and dyslipidemia, respectively. In addition, there were no statistically significant differences between the two groups regarding their medical history.

**Table 2:** Distribution of the patients regarding medical history SD = standard deviation; BMI = body mass index; kg/m2 = kilogram per squared meters; OSAS = obstructive sleep apnea syndrome; GERD = gastroesophageal reflux disease

Item	Sleeve gastrectomy (n=50)	Roux-en-Y gastric bypass (n=50)	p-value
BMI (kg/m^2^), mean±sd	43.90 ± 5.78	42.73 ± 5.12	0.064
Hypertension	16 (32%)	10 (20%)	0.127
Diabetes type 2	10 (20%)	13 (26%)	0.318
Dyslipidemia	10 (20%)	4 (8%)	0.074
Joint pain	20 (40%)	15 (30%)	0.201
GERD	2 (4%)	5 (10%)	0.218
OSAS	9 (18%)	8 (16%)	0.500

Table 3 reveals that the mean BMI at one year is 29.70 kg/m^2^ after sleeve compared with 28.64 kg/m^2^ after RYGB. The mean BMI at four years is 30.67 kg/m^2^ after sleeve compared with 30.32 kg/m^2^ after RYGB. The difference is 0.97 in the sleeve group and 1.68 in RYGB after four years. Meanwhile, the difference in baseline vs four years is 13.23 in the SG group and 12.41 in the RYGB group.

**Table 3:** Comparison between BMI of sleeve gastrectomy and Roux-en-Y gastric bypass groups *Significance P value < 0.05; **high significance P value < 0.01

Items	Sleeve gastrectomy (n=50)	Roux-en-Y gastric bypass (n=50)	p-value
	BMI after 1 year	29.70	28.64	0.04^*^
	BMI after 4 years	30.67	30.32	0.001^**^
Difference 1vs 4 years	0.97	1.68	0.001^**^
Difference baseline vs 4 years	13.23	12.41	0.226

Table 4 reveals that there were no statistically significant differences between SG and RYGB groups regarding comorbidity at follow-up of four years, while Table 5 indicates that, in the sleeve group, 10% of patients experienced minor problems within 30 days of surgery compared to 26% of those in the RYGB with (p-value=0.04). However, 26% of the sample in the sleeve group experienced major complications within 30 days of surgery compared to 16% in the RYGB group (p-value=0.05).

**Table 4:** Comparison of comorbidity at follow-up of four years between SG and RYGB groups *Significance P value < 0.05; **high significance P value < 0.01

Items	SG (n = 50)	RYGB (n = 50)	p value
DM type 2			
Comorbidity present at baseline	10 (20%)	13 (26%)	0.32
No difference	6 (12%)	4 (8%)	
Resolution	0 (0%)	5 (10%)	
Partial resolution	2 (4%)	3 (6%)	
Worse	2 (4%)	1 (2%)	
HT			
Comorbidity present at baseline	16 (32%)	10 (20%)	0.13
No difference	7 (14%)	1 (2%)	
Resolution	2 (4%)	7 (14%)	
Partial resolution	5 (10%)	2 (4%)	
Worse	2 (4%)	2 (4%)	
DL			
Comorbidity present at baseline	10 (20%)	4 (8%)	0.07
No difference	4 (8%)	4 (8%)	
Resolution	1 (2%)	2 (4%)	
Partial resolution	1 (2%)	1 (2%)	
Worse	4 (8%)	1 (2%)	
Joint pain			
Comorbidity present at baseline	20 (40%)	15 (30%)	0.20
No difference	7 (14%)	4 (8%)	
Resolution	4 (8%)	3 (6%)	
Partial resolution	2 (4%)	7 (14%)	
Worse	7 (14%)	1 (2%)	
GERD			
Comorbidity present at baseline	2 (4%)	5 (10%)	0.22
No difference	2 (4%)	2 (4%)	
Resolution	1 (2%)	2 (4%)	
Partial resolution	1 (2%)	1 (2%)	
Worse	1 (2%)	1 (2%)	
OSAS			
Comorbidity present at baseline	9 (18%)	8 (16%)	0.50
No difference	2 (4%)	3 (6%)	
Resolution	1 (2%)	2 (4%)	
Partial resolution	6 (12%)	2 (4%)	
Worse	1 (2%)	1 (2%)	

**Table 5:** Complications between groups *Significance P value ≤ 0.05; ** High significance P value < 0.01

Items	SG (n = 50)	RYGB (n = 50)	p value
Minor complication			
Vomiting	4 (8%)	3 (6%)	0.04^*^
Stricture anastomoses	0 (0%)	1 (2%)	
Swallowing disorder	1 (2%)	1 (2%)	
UTI	0 (0%)	1 (2%)	
Pain	0 (0%)	6 (12%)	
Allergic reaction	0 (0%)	1 (2%)	
Total	5 (10%)	13 (26%)	
Major complications			
Hemorrhage	4 (8%)	3 (6%)	0.05*
Leakage	5 (10%)	2 (4%)	
Infected hematoma	3 (6%)	1 (2%)	
Incisional hernia	1 (2%)	1 (2%)	
Torsion of the enteroanastomosis	0 (0%)	1 (2%)	
Procedure-related mortality	0 (0%)	0 (0%)	
Total	13 (26%)	8 (16%)	

## Discussion

In recent decades, the application of weight loss surgery as a treatment for obesity and obesity-related disorders has risen exponentially [19]. Regarding the studied patients’ sociodemographic characteristics, we found that most of the studied sample were women, with an average age of 42.02 ± 10.125 years for both groups and a mean BMI of 43.3 ± 5.45. This finding is similar to that of Hedberg et al. [20], who conducted a study in Sweden and Norway with a sample of 1,735 divided into two groups according to the type of bariatric surgery performed. They deduced that most of the sample were women, with a mean age of 42.9 ± 11.1 years and a mean BMI of 40.8 ± 3.7. The researchers' point of view that most of the studied sample was female related to many factors, such as societal factors, in which females are more seeking beauty to be thinner, also related to hormonal changes and reproductive factors where female may change her diet and her lifestyle, resulting in more weight gain. Finally, it may be in relation to stress or behavioral factors.

Similarly, a study [13], conducted in two Dutch bariatric hospitals with a five-year follow-up and a sample size of 628, divided into two groups according to the type of bariatric surgery performed, concluded that the majority of the sample were women with a mean age of 43 ± 11.1 years and a mean BMI of 43.5 ± 4.7. This agreement may be because women are more likely than men to perceive their obesity accurately and attempt weight loss but are also less likely to succeed, making them more likely to undergo weight-loss surgery.

Concerning medical history at baseline, the current research revealed that most of the studied sample in both groups suffered from joint pain, HT, DM, DL, OSAS, and GERD. The patients in the SG group mostly suffered from joint pain (40%), followed by HT (32%), whereas, in the RYGB group, they mostly suffered from joint pain (30%), followed by DM (26%). Conversely, the previous results were not consistent with the study of Oria et al. [18], who mentioned that the most common comorbidity that patients in both groups suffered from was HT, followed by DL. In a study [13], the main comorbidity was HT followed by DL in the SG group, and HT followed by joint pain in the RYGB group.

In terms of the BMI reduction following both procedures, our analysis showed no significance in both techniques. This may be due to the study limitation of a small sample size and may contributed to no differences in basal BMI, which is consistent with the findings of several studies [21-23], which also showed a greater reduction following RYGB. However, in two other studies [11,24], the researchers found no significant difference between the two procedures.

SG is primarily a restrictive procedure, achieving acceptable results in terms of weight loss during the first postoperative years [25]. Nonetheless, there is growing evidence that weight tends to return after three to five years, leading to the resurgence of obesity-related comorbidities [26]. This trend is also demonstrated in our study, which showed a rise in the average BMI four years post-surgery to roughly 30.67 kg/m² following SG, compared to 30.32 kg/m² following RYGB. However, there is no discernible difference between the two groups. This suggests that, in terms of long-term weight loss, both surgical methods are clinically equivalent.

After four years, the comorbidities associated with obesity were reduced in both groups, with variations favoring the bypass group, which achieved a slightly greater long-term resolution of T2DM, HT, DL, GERD, and joint pain compared to SG. From the researchers' point of view with RYGB, the gastrointestinal tract is altered more thoroughly, which may result in more substantial hormonal alterations that enhance metabolic function and also be due to the gut microbiota's changed composition related to metabolic health, and this will impact the glycemic control and lipid profile particularly. This finding is congruent with the evidence that RYGB is not superior to SG in terms of T2DM and HT resolution [11,27] but aligns with [13], which demonstrated statistically significant advantages in DL and GERD in RYGB over SG. Furthermore, according to the Oseberg experiment, which compared how both operations affected T2DM, RYGB is a more successful way to resolve the condition [28,29].

In this study, OSAS improved more in the SG group compared to the RYGB group after surgery. This result may be due to the method of intervention of both groups that may impact the airway, and the risk of airway collapse may be influenced by changes in upper airway muscle tone brought on by the complex surgical procedures and the weight loss that follows. This result was consistent with previous research [30] conducted by the German Bariatric Surgery Registry from 2005 to 2021, with a total sample of 2,524 patients. However, it contrasts with Biter et al. [13], which revealed that, following surgery, OSAS improved in both groups with no discernible difference between the two methods.

Regarding complications, the RYGB group was observed to have a higher complication rate (40%) than SG (36%), consistent with two studies [11,24]. However, a study [13] in Egypt, with a sample of 30 patients distributed equally between the two different surgery groups, demonstrated more complications in the SG group (20%) compared to the RYGB group (13.3%). Consistent with previous research, the most common consequence seen in the SG group was an incidence of leakage [13]. However, as the SLEEVEPASS trial also showed, patients who underwent RYGB experienced a higher rate of mild complications [11]. We found that an increase in stomach pain was mostly responsible for these mild complications. This may be explained by the significant alteration in gut anatomy, which necessitates additional dietary and lifestyle modifications. The most common reasons for patients to seek emergency medical attention are pain and dietary issues.

In interpreting the results of this study, several limitations should be acknowledged. First, the retrospective, single-center design may limit the generalizability of the findings to broader populations and different healthcare settings. The relatively small sample size of 100 patients, equally divided between the two surgical groups, may also reduce the statistical power to detect differences between the procedures, particularly for less common outcomes. Additionally, although the follow-up period extends to four years, it may not fully capture the long-term efficacy and safety of SG and RYGB, especially concerning the durability of weight loss and comorbidity resolution. The study’s reliance on patient self-reporting for certain outcomes, such as GERD symptoms, introduces the potential for recall bias. Moreover, the exclusion of patients with significant previous abdominal surgery, metabolic conditions, or those unable to adhere to follow-up protocols may have led to a selection bias, potentially skewing the results toward a healthier, more compliant cohort.

Despite these limitations, the study has several notable strengths that contribute to the robustness of its findings. The direct comparison of two widely performed bariatric procedures - SG and RYGB - provides valuable insights into their relative efficacy and safety in a well-defined patient population. The inclusion of a four-year follow-up period enhances the understanding of the long-term outcomes associated with both procedures, offering a comprehensive evaluation of their durability in weight loss and comorbidity resolution. The use of objective clinical measurements, alongside patient-reported outcomes, strengthens the reliability of the data. Furthermore, the study’s strict inclusion and exclusion criteria ensure a more homogeneous study population, reducing potential confounding variables and allowing for a clearer interpretation of the results. Additionally, the multidisciplinary approach, involving surgical, nutritional, and psychological assessments, provides a holistic evaluation of patient outcomes, underscoring the importance of comprehensive care in bariatric surgery.

## Conclusions

A frequent condition that affects millions of adults globally is obesity. A BMI higher than 30 kg/m^2^ is the definition of it. The most common bariatric procedures are the gastric bypass and laparoscopic sleeve gastrectomy, and it is effective for weight loss. The key findings of this research are as follows: first, there are no significant differences in basal BMI between the two groups before surgery and at follow-up after four years, while there were statistically significant differences between them after four years than one year after surgery, and both groups showed a significant decrease in weight and improvement in comorbidities to some extent. Second, RYGB showed superior enhancement in comorbidities such as T2DM, HT, DL, GERD, and joint pain than SG, while greater resolution of OSAS occurred in SG. Finally, RYGB showed more complications, especially minor complications such as pain, but SG showed fewer complications prominently leakage. In general, both SG and RYGB are successful surgical procedures for reducing obesity. The selection between the two operations may be influenced by the preferences, comorbidities, and unique characteristics of each patient. Future research perspectives are needed to investigate the long-term results and relative efficacy of these operations.
